# Epidemiology of periodontal disease in dogs in the UK primary‐care veterinary setting

**DOI:** 10.1111/jsap.13405

**Published:** 2021-08-09

**Authors:** D. G. O'Neill, C. E. Mitchell, J. Humphrey, D. B. Church, D. C. Brodbelt, C. Pegram

**Affiliations:** ^1^ Department of Pathobiology and Population Sciences The Royal Veterinary College Herts AL9 7TA UK; ^2^ Court Row Farm Lincolnshire NG34 9NE UK; ^3^ Department of Clinical Sciences and Services The Royal Veterinary College Herts AL9 7TA UK

## Abstract

**Objectives:**

Periodontal disease is a frequent diagnosis of dogs and can have severe negative impacts on welfare. It was hypothesised that breeds with skull shapes that differ most in conformation from the moderate mesocephalic skull shape have higher odds of periodontal disease.

**Materials and Methods:**

The cohort study included a random sample of dogs under primary veterinary care in 2016 from the VetCompass Programme database. Risk factor analysis used random effects multivariable logistic regression modelling.

**Results:**

The study included a random sample of 22,333 dogs. The 1‐year period prevalence for diagnosis with periodontal disease was 12.52% (95% CI: 12.09 to 12.97). Eighteen breeds showed increased odds compared with crossbred dogs. Breeds with the highest odds included Toy Poodle (odds ratio 3.97, 95% confidence intervals 2.21 to 7.13), King Charles Spaniel (odds ratio 2.63, 95% confidence interval 1.50 to 4.61), Greyhound (odds ratio 2.58, 95% confidence interval 1.75 to 3.80) and Cavalier King Charles Spaniel (odds ratio 2.39, 95% confidence interval 1.85 to 3.09). Four breeds showed reduced odds compared with crossbreds. Brachycephalic breeds had 1.25 times the odds (95% confidence interval 1.11 to 1.42) of periodontal disease compared with mesocephalic breeds. Spaniel types had 1.63 times the odds (95% confidence interval 1.42 to 1.87) compared with non‐spaniel types. Increasing adult bodyweight was associated with progressively decreasing odds of periodontal disease.

**Clinical Significance:**

The high prevalence identified in this study highlights periodontal disease as a priority welfare concern for predisposed breeds. Veterinarians can use this information to promote improved dental care in predisposed dogs, especially as these dogs age.

## INTRODUCTION

Periodontal disease is a syndromic diagnosis term covering the presence of at least one from a range of more specific diagnoses including gingivitis and periodontitis that exist along a continuum (Niemiec 2013, Bellows *et al*. 2019, Ruparell *et al*. 2021). Periodontal disease is one of the most frequent diagnoses made for dogs under first opinion veterinary care (Lund *et al*. 1999, O'Neill *et al*. 2014, Robinson *et al*. 2016), with some prospective studies suggesting prevalence values as high as 44% to 63.6% (Butković *et al*. 2001, Kortegaard *et al*. 2008). A recent study that scored the welfare impact of common disorders in dogs identified dental disease as having the highest overall welfare impact score from the common disorders of dogs (Summers *et al*. 2019). This finding was driven by the high prevalence (9.6%) and duration (39.9% of cases were affected for the entire study period) of periodontal disease along with a moderate severity (score of 7/21) (Summers *et al*. 2019). In addition to direct oral effects, dental disease is also associated with reduced overall systemic health (Pavlica 2008, Glickman *et al*. 2009, Bellows *et al*. 2019) including associations with disease in other organ systems such as kidneys, heart and liver (DeBowes *et al*. 1996), and even death due to starvation or secondary septicemia (de Campos Andrade *et al*. 2015, Janssens *et al*. 2016, Soe *et al*. 2017). Although gingivitis is considered to be clinically reversible, periodontitis is considered irreversible and leads to destruction of the periodontal ligament, cementum and alveola bone that often results in tooth loss (Wallis & Holcombe 2020).

Despite its high prevalence and welfare impact, there is limited published evidence on the epidemiology of periodontal disease in the wider dog population. This may be partially because many periodontal cases are managed completely in the primary care setting whereas much of the previous veterinary research literature emanated from the referral care or experimental research settings (Bartlett *et al*. 2010). Generalisability to the wider companion dog population from many of the earlier published studies on periodontal disease is limited by relatively small sample sizes of dogs that were often housed in laboratories or selected from the subset of extremely severe cases that were referred for secondary veterinary care (Butković *et al*. 2001, Marshall *et al*. 2014, Gordon *et al*. 2018). From the primary care studies that have been published, the breeds reported with frequent periodontal disease tended to be smaller sized dogs and include Yorkshire Terriers, Cocker Spaniels, West Highland White Terriers, Border Terriers and Poodles (Marshall *et al*. 2014, O'Neill *et al*. 2014, Wallis & Holcombe 2020). Medium‐ and large‐sized breeds, including Labrador Retrievers, Rottweilers, German Shepherd Dogs and Staffordshire Bull Terriers, tended to be reported with lower frequencies of periodontal disease (O'Neill *et al*. 2017a, O'Neill *et  al*. 2017c, McGreevy *et al*. 2018, Pegram *et al*. 2020). Greyhounds are a reported exception to this trend, with a recent study identifying 39% of Greyhounds under primary veterinary care in the UK as affected during a single year (O'Neill *et al*. 2019c). This prevalence in Greyhounds is more than four times higher than the 9.3% prevalence previously reported across all dog breeds (O'Neill *et al*. 2014). Until recently, the majority of epidemiological studies focused on reporting disorder predisposition (Gough *et al*. 2018). However, identification of factors associated with protection from disorders could additionally be valuable to provide deeper understanding of the aetiopathogenesis and impact of individual disorders to the health profile of a breed (Pegram *et al*. 2020, O'Neill *et al*. 2020a).

Although there is substantial published evidence linking dental health to factors such as bodyweight (prevalence is reported to decrease with increasing bodyweight) (Harvey *et al*. 1994), diet (a soft diet is considered to increase the risk) (Hill 1998, Lund *et al*. 1999) and age (incidence is reported to increase with age) (Harvey *et al*. 1994, Kortegaard *et al*. 2008, Marshall *et al*. 2014), there is limited understanding of associations between periodontal disease and body conformation. Dogs are the most phenotypically diverse companion animal species and therefore offer unique opportunities to explore correlations between conformational morphology and health (Wayne *et al*. 2006, O'Neill *et al*. 2014). The UK Kennel Club currently recognises 218 breeds from an estimated 400 breeds that exist worldwide (The Kennel Club 2021b). Skull morphology, in particular, has attracted a great deal of attention in recent years in relation to associations with several disorders (Drake & Klingenberg 2010, O'Neill *et al*. 2020b, BWG 2021) but there is currently limited evidence on associations between skull conformation and periodontal disease. A recent cross‐sectional study reported no significant association between skull morphology and severity of periodontal disease but these results may have been subject to selection bias because the study dogs were all from a commercial breeding environment and the sample size was relatively small (445 dogs) (Stella *et al*. 2018).

Given the substantial welfare harms, such as pain and systemic infections, in combination with the high frequency of periodontal disease, greater understanding of which breeds and conformational features are either predisposed to, or protected from, periodontal disease could promote more effective and targeted health approaches to mitigate these harms (Niemiec *et al*. 2020). Using anonymised veterinary clinical data from the VetCompass Programme (VetCompass 2021), this study aimed to report the prevalence of diagnosis with periodontal disease in dogs overall and within both common and commonly affected breeds. The study also aimed to report on demographic risk factors for diagnosis with periodontal disease, placing special focus on associations with breed and skull conformation. The study hypothesised that dogs with brachycephalic (broad headed) and dolichocephalic (long headed) skull conformations have higher odds of diagnosis with periodontal disease than dogs with more moderate mesocephalic skull conformation. The study did not aim to report in clinical aspects of periodontal disease in dogs.

## METHODS

### Study design

The study population included all available dogs under primary veterinary care at clinics participating in the VetCompass Programme during 2016. Dogs under veterinary care were defined as those with either (1) at least one electronic patient record (EPR) recorded during 2016 or (2) at least one EPR recorded during both 2015 and 2017. VetCompass collates de‐identified EPR data from primary‐care veterinary practices in the UK for epidemiological research (VetCompass 2021). Data fields available to VetCompass researchers include a unique animal identifier along with species, breed, date of birth, sex, neuter status, insurance and bodyweight, and also clinical information from free‐form text clinical notes, summary diagnosis terms (The VeNom Coding Group 2021) and treatment with relevant dates.

The study used a cohort design. From the overall population of dogs under veterinary care in 2016, a random sample of dogs were selected and followed in the clinical records for a 1‐year period (2016) to identify all dogs within the sample with a recorded diagnosis of periodontal disease. The case definition for periodontal disease required evidence in the clinical records that periodontal disease existed as a clinical condition at some point during 2016. The clinical decision‐making process was at the discretion of the attending veterinary surgeons. Sample size calculations estimated that 13,621 dogs would need to be assessed to estimate prevalence for a disorder that occurred in 10.0% of dogs (O'Neill *et al*. 2014) with 0.5% acceptable margin of error at a 95% confidence level from a population of 905,544 dogs (Epi Info CDC 2021). Ethics approval was obtained from the Royal Veterinary College Ethics and Welfare Committee (reference SR2018‐1652).

Breed descriptive information entered by the participating practices was cleaned and mapped to a VetCompass breed list derived and extended from the VeNom Coding breed list that included both recognised purebred breeds and also designer breed terms (The VeNom Coding Group 2021). A *purebred* variable categorised all dogs of recognisable breeds as “purebred,” dogs with contrived names generated from two or more purebred breed terms as designers and all remaining dogs with breed information as “crossbred” (The Kennel Club 2021b). Pure breeds and designer breeds that had over 300 dogs in the overall study population or had at least 20 periodontal disease cases were included in the *breed* variable as individual breeds. This *breed* variable also included a category that grouped all remaining purebreds and a category that grouped all general crossbred dogs. This approach was taken to facilitate statistical power for the individual breed analyses (Scott *et al*. 2012). Breeds were further characterised by skull shape (dolichocephalic, mesocephalic, brachycephalic, non‐purebred) and spaniel (spaniel, non‐spaniel, non‐purebred) status for analysis (Appendix S1). A *Kennel Club breed group* variable classified breeds recognised by the UK Kennel Club into their relevant breed groups (Gundog, Hound, Pastoral, Terrier, Toy, Utility and Working) and all remaining types were classified as non‐Kennel Club recognised (The Kennel Club 2021b).

Neuter and insurance status were defined by the final available EPR value. Adult bodyweight was defined as the mean of all bodyweight (kg) values recorded for each dog after reaching 18 months old and was categorised as: less than 10.0, 10.0 to less than 15.0, 15.0 to less than 20.0, 20.0 to less than 25.0, 25.0 to less than 30.0, 30.0 to less than 40.0 and at least 40.0. Mean adult bodyweight was generated for all breed‐sex combinations with adult bodyweight available for at least 100 dogs in the overall study population and used to categorise individual dogs as “at or above the breed‐sex mean,” “below the breed‐sex mean” and “no recorded bodyweight”. Age (years) was defined at December 31, 2016, and was categorised as: up to 1.0, 1.0 to less than 2.0, 2.0 to less than 4.0, 4.0 to less than 6.0, 6.0 to less than 8.0, 8.0 to less than 10.0, 10.0 to less than 12.0 and at least 12.0.

The list of unique animal identification numbers was randomly ordered and the clinical records of a randomly selected subset of animals were reviewed manually in detail to identify all dogs that met the case definition for periodontal disease. No distinction was made between pre‐existing and incident cases of periodontal disease. Following internal validity checking and data cleaning in Excel (Microsoft Office Excel 2013, Microsoft Corp.), analyses were conducted using Stata Version 13 (Stata Corporation).

### Statistical analysis

One‐year period prevalence values with 95% confidence intervals (CI) described the probability of diagnosis at least once during 2016 in dogs overall and in common breeds. The CI estimates were derived from standard errors based on approximation to the binomial distribution (Kirkwood & Sterne 2003). Risk factor analysis included dogs with periodontal disease as cases while all remaining dogs in the sample were classed as non‐cases. Binary logistic regression modelling was used to evaluate univariable associations between risk factors and periodontal disease during 2016. Because breed was a factor of primary interest for the study, variables that derived from the breed information and therefore were highly collinear with breed (*skull shape, spaniel, purebred, Kennel Club recognised breed* and *Kennel Club breed group*) were excluded from initial breed multivariable modelling. Instead, each of these variables individually replaced the *breed* variable in the main final breed‐focused model to evaluate their effects after taking account of the other variables. *Adult bodyweight* (a defining characteristic of individual breeds) replaced breed and *bodyweight relative to breed‐sex mean* in the final breed‐focused model. Risk factors with liberal associations in univariable modelling (P<0.2) were taken forward for multivariable evaluation. Model development used manual backwards stepwise elimination. Model building began with a full model using the relevant variables taken forward from the univariable analysis. Variables were sequentially removed and tested for their contribution using the likelihood ratio test (P<0.05) to decide on retention. All removed variables were checked in the final model to assess for confounding effect. Pair‐wise interaction effects were evaluated for the final model variables (Dohoo *et al*. 2009). Clinic attended was evaluated as a random effect. The area under the ROC curve and the Hosmer‐Lemeshow test were used to evaluate the quality of the model fit and discrimination (non‐random effect model) (Dohoo *et al*. 2009, Hosmer *et al*. 2013). Statistical significance was set at *P*<0.05. Univariable odds ratios (ORs) are reported as OR whereas multivariable ORs are reported as adjusted odds ratio (aOR).

## RESULTS

### Prevalence

**Fig 1:**
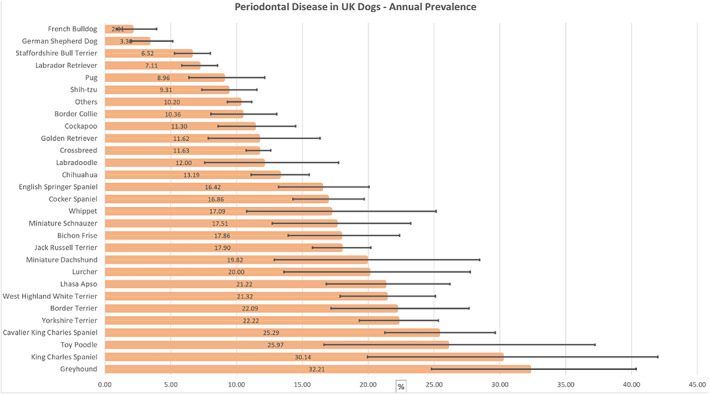
One‐year (2016) period prevalence of periodontal disease in common dog breeds under primary veterinary care in the UK. N = 22,333

The study included a random sample of 22,333 dogs attending 784 veterinary clinics from an overall population of 905,554 dogs under veterinary care in 2016. There were 2,797 of 22,333 dogs diagnosed with periodontal disease during 2016, yielding a 1‐year period prevalence of 12.52% (95% CI: 12.09 to 12.97). The breeds with the highest periodontal disease prevalence were Greyhound (32.21%, 95% CI 24.8 to 40.35), King Charles Spaniel (30.14%, 95% CI 19.94 to 42), Toy Poodle (25.97%, 95% CI 16.64 to 37.23), Cavalier King Charles Spaniel (25.29%, 95% CI 21.27 to 29.65), Yorkshire Terrier (22.22%, 95% CI 19.32 to 25.34) and Border Terrier (22.09%, 95% CI 17.18 to 27.66). The breeds with the lowest periodontal disease prevalence were Pug (8.96%, 95% CI 6.39 to 12.14), Labrador Retriever (7.11%, 95% CI 5.85 to 8.55), Staffordshire Bull Terrier (6.52%, 95% CI 5.3 to 8), German Shepherd Dog (3.30%, 95% CI 1.97 to 5.16) and French Bulldog (2.01%, 95% CI 0.87 to 3.92) (Fig. 1).

Data completeness varied between the variables assessed: breed 99.68%, age 98.80%, sex 99.66%, neuter 99.66% and adult bodyweight 66.82%. The median age of dogs with periodontal disease (7.54 years, interquartile range (IQR) 5.00 to 10.50, range 0.53 to 19.27) was older than dogs without periodontal disease (3.90 years, IQR 1.65 to 7.48, range 0.01 to 20.46) (P<0.001). The median adult bodyweight of dogs with periodontal disease (10.33 kg, IQR 7.20 to 19.33, range 1.49 to 65.94) was lighter than for dogs without periodontal disease (14.90 kg, IQR 8.37 to 25.86, range 1.41 to 85.00) (P<0.001). Further demographic information is available in Tables 1 and 2.

**Table 1:** Descriptive and univariable logistic regression (clinic attended included as a random effect) results for *breed‐related risk factors* evaluated for periodontal disease during 2016 in dogs under primary veterinary care in the VetCompass Programme in the UK

Variable	Category	Case number (%)	Non‐case number (%)	Odds ratio	95% CI	Category P‐value	Variable P‐value
Purebred status	Crossbred	548 (19.61)	4162 (21.38)	Base			0.016
	Designer	140 (5.01)	1155 (5.93)	0.90	0.74 to 1.10	0.316	
	Purebred	2107 (75.38)	14,149 (72.69)	1.11	1.01 to 1.24	**0.038**	
Breed	Crossbreed	548 (19.59)	4162 (21.30)	1.00	0 to 0		<0.001
	Greyhound	48 (1.72)	101 (0.52)	3.82	2.62 to 5.56	**<0.001**	
	King Charles Spaniel	22 (0.79)	51 (0.26)	3.35	1.97 to 5.69	**<0.001**	
	Toy Poodle	20 (0.72)	57 (0.29)	3.04	1.78 to 5.21	**<0.001**	
	Cavalier King Charles Spaniel	110 (3.93)	325 (1.66)	2.66	2.08 to 3.39	**<0.001**	
	Yorkshire Terrier	170 (6.08)	595 (3.05)	2.18	1.79 to 2.66	**<0.001**	
	Lhasa Apso	66 (2.36)	245 (1.25)	2.14	1.59 to 2.89	**<0.001**	
	Border Terrier	57 (2.04)	201 (1.03)	2.08	1.51 to 2.85	**<0.001**	
	West Highland White Terrier	110 (3.93)	406 (2.08)	2.07	1.63 to 2.62	**<0.001**	
	Lurcher	27 (0.97)	108 (0.55)	1.97	1.26 to 3.08	**0.003**	
	Bichon Frise	60 (2.15)	276 (1.41)	1.68	1.24 to 2.28	**0.001**	
	Jack Russell Terrier	213 (7.62)	977 (5.00)	1.66	1.39 to 1.98	**<0.001**	
	Miniature Schnauzer	38 (1.36)	179 (0.92)	1.63	1.12 to 2.37	**0.010**	
	Whippet	20 (0.72)	97 (0.50)	1.63	0.98 to 2.70	0.058	
	Miniature Dachshund	22 (0.79)	89 (0.46)	1.60	0.97 to 2.62	0.064	
	Cocker Spaniel	130 (4.65)	641 (3.28)	1.50	1.21 to 1.87	**<0.001**	
	English Springer Spaniel	78 (2.79)	397 (2.03)	1.49	1.14 to 1.95	**0.003**	
	Chihuahua	126 (4.50)	829 (4.24)	1.16	0.94 to 1.44	0.164	
	Labradoodle	21 (0.75)	154 (0.79)	0.98	0.61 to 1.59	0.948	
	Cockapoo	54 (1.93)	424 (2.17)	0.94	0.69 to 1.27	0.681	
	Golden Retriever	28 (1.00)	213 (1.09)	0.91	0.60 to 1.38	0.655	
	Border Collie	63 (2.25)	545 (2.79)	0.87	0.65 to 1.15	0.322	
	Others	440 (15.73)	3872 (19.82)	0.84	0.73 to 0.96	**0.012**	
	Shih‐tzu	74 (2.65)	721 (3.69)	0.82	0.63 to 1.07	0.140	
	Pug	37 (1.32)	376 (1.92)	0.73	0.51 to 1.04	0.080	
	Labrador Retriever	104 (3.72)	1358 (6.95)	0.55	0.44 to 0.69	**<0.001**	
	Staffordshire Bull Terrier	85 (3.04)	1219 (6.24)	0.52	0.41 to 0.66	**<0.001**	
	German Shepherd Dog	18 (0.64)	528 (2.70)	0.25	0.15 to 0.40	**<0.001**	
	French Bulldog	8 (0.29)	390 (2.00)	0.15	0.07 to 0.30	**<0.001**	
Kennel Club Breed Group	Not KC‐recognised	728 (26.05)	5711 (29.34)	Base			<0.001
	Toy	587 (21.00)	2726 (14.00)	1.71	1.51 to 1.93	**<0.001**	
	Hound	142 (5.08)	656 (3.37)	1.68	1.37 to 2.05	**<0.001**	
	Terrier	529 (18.93)	3119 (16.02)	1.32	1.17 to 1.49	**<0.001**	
	Utility	291 (10.41)	2227 (11.44)	1.03	0.89 to 1.2	0.649	
	Gundog	374 (13.38)	2911 (14.95)	0.97	0.84 to 1.11	0.633	
	Pastoral	97 (3.47)	1228 (6.31)	0.60	0.48 to 0.75	**<0.001**	
	Working	47 (1.68)	888 (4.56)	0.41	0.3 to 0.55	**<0.001**	
Skull conformation	Dolichocephalic	238 (8.52)	1506 (7.74)	Base			0.004
	Brachycephalic	485 (17.35)	3684 (18.94)	0.85	0.72 to 1.01	0.069	
	Crossbred	688 (24.62)	5306 (27.28)	0.83	0.71 to 0.98	**0.026**	
	Mesocephalic	1384 (49.52)	8957 (46.04)	0.97	0.84 to 1.13	0.734	
Spaniel	Non‐spaniel type	1887 (67.47)	13,804 (70.66)	1.00			<0.001
	Spaniel type	360 (12.87)	1500 (7.68)	1.79	1.57 to 2.03	**<0.001**	
	Non‐purebred	550 (19.66)	4232 (21.66)	0.97	0.87 to 1.07	0.526	

Column percentages shown in brackets

CI Confidence interval

**Table 2:** Descriptive and univariable logistic regression (clinic attended included as a random effect) results for *non‐breed‐related risk factors* evaluated for periodontal disease during 2016 in dogs under primary veterinary care in the VetCompass Programme in the UK

Variable	Category	Case number (%)	Non‐case number (%)	Odds ratio	95% CI	Category P value	Variable P value
Adult (>18 months) bodyweight (kg)	< 10 kg	1114 (47.34)	4300 (34.21)	2.43	2.04 to 2.88	**<0.001**	
	10.0 to < 15.0	459 (19.51)	2005 (15.95)	2.13	1.77 to 2.58	**<0.001**	
	15.0 to < 20.0	219 (9.31)	1475 (11.73)	1.35	1.09 to 1.68	**0.006**	
	20.0 to < 25.0	194 (8.24)	1410 (11.22)	1.24	1.00 to 1.55	0.054	
	25.0 to < 30.0	152 (6.46)	1198 (9.53)	1.15	0.91 to 1.46	0.235	
	30.0 to < 40.0	176 (7.48)	1593 (12.67)	Base			<0.001
	> or = 40	39 (1.66)	590 (4.69)	0.61	0.42 to 0.87	**0.007**	
	< 10	1114 (47.34)	4300 (34.21)	2.43	2.04 to 2.88	**<0.001**	
Bodyweight relative to breed mean	At or above	1027 (36.72)	5801 (29.69)	Base			<0.001
	Below	1322 (47.26)	6724 (34.42)	1.11	1.01 to 1.21	**0.030**	
	Unrecorded	448 (16.02)	7011 (35.89)	0.28	0.24 to 0.32	**<0.001**	
Age (years)	< 1.0	9 (0.32)	2499 (12.79)	0.04	0.02 to 0.08	**<0.001**	
	1.0 to < 2.0	70 (2.5)	3208 (16.42)	0.24	0.18 to 0.31	**<0.001**	
	2.0 to < 4.0	369 (13.19)	4089 (20.93)	Base			<0.001
	4.0 to < 6.0	516 (18.45)	2937 (15.03)	1.97	1.71 to 2.28	**<0.001**	
	6.0 to < 8.0	546 (19.52)	2252 (11.53)	2.75	2.38 to 3.18	**<0.001**	
	8.0 to < 10.0	481 (17.2)	1767 (9.04)	3.06	2.64 to 3.56	**<0.001**	
	10.0 to < 12.0	365 (13.05)	1208 (6.18)	3.51	2.98 to 4.13	**<0.001**	
	> or = 12.0	425 (15.19)	1325 (6.78)	3.58	3.06 to 4.19	**<0.001**	
	Unrecorded	16 (0.57)	251 (1.28)	0.74	0.44 to 1.24	0.250	
Sex/neuter	Female entire	435 (15.55)	5249 (26.87)	Base			<0.001
	Female neutered	868 (31.03)	3988 (20.41)	2.57	2.27 to 2.92	**<0.001**	
	Male entire	606 (21.67)	5871 (30.05)	1.22	1.07 to 1.39	**0.003**	
	Male neutered	883 (31.57)	4358 (22.31)	2.39	2.11 to 2.71	**<0.001**	
	Unrecorded	5 (0.18)	70 (0.36)	0.87	0.35 to 2.2	0.774	
Insurance	Uninsured	2268 (81.09)	17,086 (87.46)	Base			<0.001
	Insured	529 (18.91)	2450 (12.54)	1.42	1.27 to 1.60	**<0.001**	

Column percentages shown in brackets

CI Confidence interval

### Risk factors

All tested variables were liberally associated with diagnosis with periodontal disease in univariable logistic regression modelling and were evaluated using multivariable logistic regression modelling as described in the methods (Tables 1 and 2). The final main breed‐focused multivariable model retained five risk factors: *breed, bodyweight relative to breed‐sex mean*, *age, sex‐neuter* and *insurance* (Table 3). The final model was improved by inclusion of the clinic attended as a random effect (rho: 0.01 indicating that 1% of the variability was accounted for by the clinic attended, P=0.001). The final random effects model showed acceptable model‐fit (Hosmer‐Lemeshow test statistic: P=0.231) and acceptable discrimination (area under the ROC curve: 0.767).

**Table 3:** Final breed‐focused random effects multivariable logistic regression model for risk factors associated with periodontal disease in dogs under primary veterinary care in the VetCompass Programme in the UK

Variable	Category	Odds ratio	95% CI	Category P‐value	Variable P‐value
Breed	Crossbreed	Base			<0.001
	Toy Poodle	3.97	2.21 to 7.13	**<0.001**	
	King Charles Spaniel	2.63	1.50 to 4.61	**0.001**	
	Greyhound	2.58	1.75 to 3.80	**<0.001**	
	Cavalier King Charles Spaniel	2.39	1.85 to 3.09	**<0.001**	
	Lhasa Apso	2.24	1.63 to 3.06	**<0.001**	
	Yorkshire Terrier	2.16	1.75 to 2.67	**<0.001**	
	Cockapoo	2.11	1.51 to 2.94	**<0.001**	
	Chihuahua	2.09	1.66 to 2.63	**<0.001**	
	Lurcher	1.93	1.21 to 3.10	**0.006**	
	Border Terrier	1.85	1.32 to 2.58	**<0.001**	
	Miniature Dachshund	1.80	1.06 to 3.06	**0.029**	
	Whippet	1.74	1.02 to 2.98	**0.042**	
	Bichon Frise	1.67	1.21 to 2.30	**0.002**	
	Cocker Spaniel	1.66	1.32 to 2.09	**<0.001**	
	Miniature Schnauzer	1.66	1.12 to 2.46	**0.012**	
	West Highland White Terrier	1.47	1.15 to 1.88	**0.002**	
	Jack Russell Terrier	1.37	1.14 to 1.65	**0.001**	
	English Springer Spaniel	1.33	1.00 to 1.75	**0.047**	
	Labradoodle	1.26	0.77 to 2.08	0.363	
	Pug	1.17	0.81 to 1.71	0.405	
	Others	0.96	0.83 to 1.10	0.538	
	Shih‐tzu	0.92	0.70 to 1.20	0.523	
	Golden Retriever	0.81	0.53 to 1.25	0.346	
	Border Collie	0.77	0.57 to 1.03	0.074	
	Labrador Retriever	0.49	0.39 to 0.62	**<0.001**	
	Staffordshire Bull Terrier	0.45	0.35 to 0.58	**<0.001**	
	French Bulldog	0.43	0.21 to 0.88	**0.022**	
	German Shepherd Dog	0.25	0.15 to 0.40	**<0.001**	
Bodyweight relative to breed mean	At or above	Base			<0.001
	Below	1.31	1.19 to 1.44	**<0.001**	
	Unrecorded	0.62	0.54 to 0.72	**<0.001**	
Age (years)	< 1.0	0.06	0.03 to 0.12	**<0.001**	<0.001
	1.0 to < 2.0	0.30	0.23 to 0.39	**<0.001**	
	2.0 to < 4.0	Base			
	4.0 to < 6.0	1.91	1.65 to 2.22	**<0.001**	
	6.0 to < 8.0	2.76	2.37 to 3.21	**<0.001**	
	8.0 to < 10.0	3.17	2.71 to 3.71	**<0.001**	
	10.0 to < 12.0	3.76	3.17 to 4.46	**<0.001**	
	> or = 12.0	3.91	3.31 to 4.61	**<0.001**	
	Unrecorded	1.34	0.77 to 2.32	0.294	
Sex/neuter	Female entire	Base			<0.001
	Female neutered	1.41	1.24 to 1.62	**<0.001**	
	Male entire	1.19	1.03 to 1.37	**0.016**	
	Male neutered	1.37	1.20 to 1.57	**<0.001**	
	Unrecorded	1.39	0.51 to 3.77	0.522	
Insurance	Uninsured	Base			<0.001
	Insured	1.30	1.14 to 1.47	**<0.001**	

Clinic attended was included as a random effect. N=22,333

CI Confidence interval

After accounting for the effects of the other variables evaluated, 18 breed types showed increased adjusted odds of periodontal disease compared with crossbred dogs. The breeds with the highest adjusted odds included Toy Poodle (aOR 3.97, 95% CI 2.21 to 7.13, P<0.001), King Charles Spaniel (aOR 2.63, 95% CI 1.50 to 4.61, P=0.001), Greyhound (aOR 2.58, 95% CI 1.75 to 3.80, P<0.001) and Cavalier King Charles Spaniel (aOR 2.39, 95% CI 1.85 to 3.09, P<0.001). Four breeds showed reduced adjusted odds of periodontal disease compared with crossbreds: German Shepherd Dog (aOR: 0.25, 95% CI 0.15 to 0.40, P<0.001), French Bulldog (aOR: 0.43, 95% CI 0.21 to 0.88, P=0.022), Staffordshire Bull Terrier (aOR: 0.45, 95% CI 0.35 to 0.58, P=0.001) and Labrador Retriever (aOR: 0.49, 95% CI 0.39 to 0.62, P=0.001).

Dogs with an adult bodyweight below their breed‐sex mean had 1.31 (95% CI 1.19 to 1.44, *P*<0.001) times the adjusted odds of periodontal disease compared with dogs that weighed at or above their breed‐sex mean. Increasing age was associated with progressively increasing adjusted odds of periodontal disease. Entire females had lower adjusted odds of periodontal disease compared with the other sex/neuter combinations. Insured dogs had 1.30 (95% CI 1.14 to 1.47, P<0.001) times the adjusted odds of periodontal disease compared with uninsured dogs (Table 3).

As described in the methods, some variables were used to replace the breed variable in the final breed‐focused model. Designer types had 1.63 times the adjusted odds (95% CI 1.31 to 2.02, P<0.001) of periodontal disease compared with crossbred dogs. Toy (1.89 aOR, 95% CI 1.66 to 2.15, P<0.001) and Hound (1.52 aOR, 95% CI 1.23 to 1.89, P<0.001) Kennel Club breed groups showed higher adjusted odds of periodontal disease compared with breeds that are not recognised by the Kennel Club. Spaniel types had 1.63 times the adjusted odds (95% CI 1.42 to 1.87, P<0.001) of periodontal disease compared with non‐spaniel types. Increasing adult bodyweight was associated with progressively decreasing adjusted odds of periodontal disease. Brachycephalic dog types had 1.25 times the adjusted odds (95% CI 1.11 to 1.42, P<0.001) of periodontal disease compared with mesocephalic types (Table 4).

**Table 4:** Results for risk factors that directly replaced the breed variable in the final breed‐focused random effects multivariable logistic regression model (along with age, bodyweight relative to breed mean, sex/neuter and insurance status)

Variable	Category	Odds ratio	95% CI	Category P‐value	Variable P‐value
Purebred status	Crossbred	Base			<0.001
	Designer	1.63	1.31 to 2.02	<0.001	
	Purebred	1.13	1.02 to 1.26	0.023	
Kennel Club Breed Group	Not KC‐recognised	1.00	0 to 0		<0.001
	Toy	1.89	1.66 to 2.15	<0.001	
	Hound	1.52	1.23 to 1.89	<0.001	
	Utility	1.16	0.99 to 1.36	0.061	
	Terrier	0.98	0.86 to 1.12	0.775	
	Gundog	0.81	0.7 to 0.93	0.003	
	Pastoral	0.49	0.39 to 0.62	<0.001	
	Working	0.39	0.28 to 0.53	<0.001	
Skull conformation	Mesocephalic	Base			0.010
	Brachycephalic	1.25	1.11 to 1.42	<0.001	
	Dolichocephalic	1.08	0.92 to 1.26	0.363	
	Non‐purebred	1.02	0.92 to 1.13	0.704	
Spaniel	Non‐spaniel type	Base			<0.001
	Spaniel type	1.63	1.42 to 1.87	<0.001	
	Non‐purebred	0.92	0.82 to 1.02	0.111	
Adult (>18 months) bodyweight (kg)	< 10.0	3.07	2.57 to 3.67	<0.001	<0.001
	10.0 to < 15.0	2.48	2.04 to 3.02	<0.001	
	15.0 to < 20.0	1.50	1.20 to 1.87	<0.001	
	20.0 to < 25.0	1.29	1.03 to 1.62	0.027	
	25.0 to < 30.0	1.23	0.97 to 1.56	0.095	
	30.0 to < 40.0	Base			
	≥ 40.0	0.58	0.40 to 0.84	0.004	
	Unavailable	1.07	0.87 to 1.31	0.526	

Adult (>18 months) bodyweight (kg) replaced the breed and bodyweight relative to breed mean variables in the final breed‐focused random effects multivariable logistic regression model. These results report associations between these risk factors and periodontal disease in dogs under primary veterinary care in the VetCompass Programme in the UK. Clinic attended was included as a random effect. N=22,333

CI Confidence interval

## DISCUSSION

This paper is part of a new research paradigm that goes beyond previous research tendency to focus primarily on predisposition (Gough *et al*. 2018). Instead, the study design has been extended to explore breeds and conformations that are either predisposed to, or protected from periodontal disease, in order to provide evidence that can support moves to select towards positive features as well as away from negative features (Pegram *et al*. 2020, O'Neill *et al*. 2020a, The Kennel Club 2021a).

The current study reports a 1‐year period prevalence of 12.52% for periodontal disease diagnosis within the general dog population under primary veterinary care. This result is in line with other retrospective studies of individual breeds based on primary care clinical data (O'Neill *et al*. 2017a, O'Neill *et al*. 2017b, O'Neill *et al*. 2017c, McGreevy *et al*. 2018, O'Neill *et al*. 2018, O'Neill *et al*. 2019a, O'Neill *et al*. 2019b, O'Neill *et al*. 2020b) but is substantially lower than reported in several prospective studies that reported prevalence as high as 63% (Butković *et al*. 2001, Kortegaard *et al*. 2008). This difference may result from a lower inclusion clinical threshold and more rigorous dental examinations within prospective studies and suggests that many true cases of periodontal disease may be missed in the primary care setting. In many dogs under first opinion care, dental health status is determined based on clinical examination alone whereas more in‐depth diagnostic methods such as full‐mouth radiography have been reported to identify additional dental disease lesions that were not noted on routine oral examination, with this difference being more noteworthy in older dogs (Kim *et al*. 2013). A study of 114 dogs diagnosed with active periodontal disease on full‐mouth examination under anaesthesia identified that only 82% of these dogs showed inflammation on visual examination while conscious (Queck *et al*. 2018). Newer methods such as the visual dental scale (Bauer *et al*. 2018), assessment of canine gingival margin plaque (Ruparell *et al*. 2021) and the thiol‐detection test (Queck *et al*. 2018) offer opportunities for clinicians to improve diagnostic rates for periodontal disease in the first opinion setting. It is noteworthy though that many prospective studies of periodontal disease prevalence relied on targeted selection of study animals, often in laboratory‐like environments or referral setting, which may reduce the generalisability of these findings to the wider dog population (Marshall *et al*. 2014, Stella *et al*. 2018, Wallis *et al*. 2018, Pereira dos Santos *et al*. 2019). In contrast, the current study included a random sample of dogs under primary veterinary care within the VetCompass Programme and should therefore be more representative of the general population of dogs (Bateson 2010). A study using primary care veterinary data from the USA reported higher dental disease prevalence than the current study, with 20.5% of dogs recorded with dental calculus and 19.5% recorded with gingivitis (Lund *et al*. 1999). None the less, the prevalence of 12.5% reported in the current study highlights periodontal disease as one of the most common disorders of dogs overall, with even more concerningly high prevalence values for many of the predisposed breeds highlighted. This high general prevalence exemplifies the significance of dental disease to general practice care and highlights the value of increasing the coverage of dental training in current veterinary undergraduate and postgraduate teaching (Anderson *et al*. 2017).

Periodontal disease prevalence tends to increase with age (Hamp *et al*. 1984, Harvey *et al*. 1994, Kyllar & Witter 2005, Kortegaard *et al*. 2008), with some authors suggesting that almost all dogs over the age of five years have some degree of periodontal disease (Hoffmann & Gaengler 1996). In line with this ageing effect, the median age of dogs in the current study with periodontal disease (7.54 years) was substantially higher than that of dogs without periodontal disease (3.90 years). The adjusted odds of periodontal disease also rose steeply with ageing by a factor of over 65 times; ranging from 0.06 times the adjusted odds in dogs aged under 1 year to 3.91 times the adjusted odds in dogs aged over 12 years, compared with dogs aged two to less than 4 years. With age being such an influential factor, veterinarians should be especially rigorous in assessing dental health during routine examinations in older dogs to promote earlier diagnosis and intervention. Greater emphasis on monitoring of dental health by veterinarians from puppyhood onwards, and discussion of at‐home dental care with owners, might help to reduce the welfare burden of periodontal disease later in life and thus improve the dog's quality of life (Roudebush *et al*. 2005).

Periodontal disease has long been linked to body size in dogs, with smaller breeds reportedly at greater risk than larger breeds (Harvey *et al*. 1994, Hoffmann & Gaengler 1996, Butković *et al*. 2001, Stella *et al*. 2018). Dental calculus has been reported in some small‐breed dogs as young as 1 year of age (Kyllar & Witter 2005). Breeds such as Yorkshire Terrier, Toy Poodle, Cocker Spaniel and Jack Russell Terrier are often cited as predisposed (Hamp *et al*. 1984, O'Neill *et al*. 2014). In the current study, the median adult bodyweight of dogs with periodontal disease (10.33 kg) was over 4 kg lighter than dogs without periodontal disease (14.90 kg). Dogs weighing under 10 kg had more than three times the adjusted odds of periodontal disease compared with dogs weighing 30 to less than 40 kg. In addition to any intrinsic genetic susceptibility, increased periodontal disease in smaller dogs may be associated with the challenges related to brushing the teeth of very small dogs, greater reluctance in smaller dogs to accept dental chews and a reputation for fussy eating habits (Mateo *et al*. 2020). Increased owner awareness of periodontal disease in smaller breeds should be promoted to ensure that effective preventative measures, such as tooth brushing, are put in place to reduce the likelihood of the onset of periodontal disease. It would also be valuable to explore reasons for non‐compliance by owners with current veterinary advice on routine tooth brushing in an effort to increase future compliance.

Breeds with the highest adjusted odds of periodontal disease in the current study included Toy Poodle, King Charles Spaniel, Greyhound, Cavalier King Charles Spaniel, Lhasa Apso and Yorkshire Terrier. Apart from the Greyhound, these are all small sized breeds. Conversely, many of the breeds with the lowest adjusted odds in the current study tended to be larger sized breeds, including Labrador Retriever, Staffordshire Bull Terrier and German Shepherd Dog. In an earlier study based on primary care clinical data, the least affected breeds were Border Collies (6.7% prevalence), German Shepherd Dogs (4.5%), Labrador Retrievers (3.2%) and Staffordshire Bull Terriers (2.4%) (O'Neill *et al*. 2014). Although the precise prevalence values in those studies differ slightly from the current study (Labrador Retriever 7.11%, German Shepherd Dog 3.30%, Staffordshire Bull Terrier 6.52%), these breeds are still seen to have a lower odds of periodontal disease diagnosis in the current study and could therefore be categorised as protected breeds. Dog skulls are generally classified into three distinct categories based on head shape profiles: dolichocephalic (“long‐headed”), mesocephalic (“middle‐headed”) and brachycephalic (“broad‐headed”), although there is ongoing debate on the precise allocation of breeds to each of the three skull categories (O'Neill *et al*. 2015, O'Neill *et al*. 2020a). Staffordshire Bull Terriers and Labrador Retrievers are almost universally categorised within the mesocephalic group, whereas German Shepherd Dogs are variously categorised as either mesocephalic or dolichocephalic. However, all three could be accepted to represent a skull shape with a more moderate conformation compared to the elongated skull of a Borzoi or the flattened brachycephalic faces of breeds such as Pugs and French Bulldogs (The Kennel Club 2021b). To date, much of the research on the effects of skull shape on the health of brachycephalic dog breeds has focused on respiratory disorders, neurological disease and ocular disease (Koch *et al*. 2003, O'Neill *et al*. 2015, Packer *et al*. 2015, Gordon *et al*. 2017, Liu *et al*. 2017, Knowler *et al*. 2019). However, there is also some evidence that brachycephalic breeds may also be predisposed to incisor overcrowding and that smaller breeds of dog are more susceptible to periodontal disease (Lund 2008, Burns 2016, Bellows *et al*. 2019). Dental occlusion varies considerably across skull shapes and, in extreme cases, the lower incisors can be up to 5 cm in front of the upper row (Emily & Penman 1994).

Although our results support a general predisposition for periodontal disease in brachycephalic dog types (1.25 times the adjusted odds compared with mesocephalic types), this effect was not universal across all brachycephalic breeds. French Bulldogs had a low prevalence of periodontal disease (just 2.01%) that is in line with the results from an earlier study on French Bulldogs based on UK primary‐care that did not even identify periodontal disease among the 26 most common disorders in French Bulldogs (O'Neill *et al*. 2018). Even after controlling for the effects of confounding from factors such as age, the current study still identified French Bulldogs as significantly protected to periodontal disease compared with crossbreds, showing just 0.43 times the adjusted odds. Evidence suggestive that this periodontal protective effect may be real in French Bulldogs comes from the results in the current study for the Pug, another small‐stature breed with extreme brachycephaly that has become very popular over the past 20 years (The Kennel Club 2021c). Although also featuring among the breeds with the lowest prevalence of periodontal disease (8.96%), the prevalence for Pugs was not nearly as low as the French Bulldog and, after controlling for confounding, there was no strong evidence of predisposition to periodontal disease in Pugs (aOR 1.17, 95% CI 0.81 to 1.71). A protective effect for periodontal disease in French Bulldogs could be an artefact influenced by diagnostic differences peculiar to this breed but such effects would also be expected to apply to other similar breeds such as the Pug. It is possible that high awareness of the presence of other health issues and concerns about anaesthetic risk in this breed reduce the primary care focus on routine dental examination. The relative youthfulness of the French Bulldog population compared with dogs overall may also lead to some residual confounding in the multivariable modelling (O'Neill *et al*. 2018). Equally, French Bulldogs may be truly protected to periodontal disease from effects such as breed‐related differences in food and chew stick manipulation such that French Bulldogs may benefit more from dental chews than other breeds. There is therefore scope for further research into chew efficacy in relation to breed (Mateo *et al*. 2020).

Although dolichocephaly was not associated with an overall increased adjusted odds of periodontal disease, certain dolichocephalic breeds such as the Greyhound (aOR 2.58) had higher adjusted odds of dental disease than other types of dog. A recent VetCompass study reported periodontal disease as the most prevalent disorder in Greyhounds (39% prevalence) (O'Neill *et al*. 2019c), which is slightly higher than the 32.21% prevalence reported in the current study, and supports a view that dental care should be a priority issue for Greyhounds. Predisposition to periodontal disease in Greyhounds may result in part because many of the Greyhounds seen in primary care settings are retirees from racing where maintenance of good of dental hygiene may not be deemed a priority for a working animal (EFRA 2016). Greyhound trainers often formulate their own diets composed predominantly of raw meat, which may not offer nutritional balance and could be deficient in vitamins and minerals, thus affecting dental hygiene (Hill 1998). The dolichocephalic shape of a Greyhound's skull could also put it at higher risk. With the elongation of the jaw, gaps between teeth could form, exposing more gum and allowing more food to build up between teeth. It is also possible that genetic factors are involved.

This is one of the first studies to explore periodontal disease occurrence in so‐called designer dog types, which are hybrids between differing parental purebred breeds. The results showed that designer breeds had 1.63 times the odds of diagnosis with periodontal disease compared with crossbreds and 1.44 times the odds compared with purebreds. However, the high level of periodontal disease identified in these designer types may be less to do with being designer per se and more to do with the poodle component that is common within many designer types such as the Cockapoo, Cavapoo and Labradoodle. Poodles are considered to be at high risk of periodontal disease (Hoffmann & Gaengler 1996, Dias *et al*. 2021), with the Toy Poodle identified as the breed with the highest odds of periodontal disease in the current study. These current results provide some evidence against a hybrid vigour effect in dogs that, if present, should have offered a protective effect against a polygenic disorder such as periodontal disease in these designer breeds (Nicholas *et al*. 2016).

The study had some limitations. Periodontal disease cases were defined based on a binary classification as cases or non‐cases but without further criteria such as severity, chronicity, clinical signs and diagnostics being considered in the modelling. As discussed above, it is possible that some dogs with true periodontal disease were not diagnosed clinically and may therefore have been omitted from our cases, leading to an underestimate of the true prevalence (Kim *et al*. 2013, Bauer *et al*. 2018, Queck *et al*. 2018). The specific type of dental disease is not always recorded in the clinical records and there are often inconsistencies in recording of the severity grading between veterinarians. Consequently, it was not always clear whether the clinical records referred to gingivitis (reversible) or periodontitis (irreversible) (Bauer *et al*. 2018), and therefore this distinction was not reported in the current study. The current study included clinical data gathered from a convenience sample of UK first opinion veterinary practices that participate within the VetCompass Programme. This may have selectively biased participation and therefore also the results. However, these effects should be somewhat mitigated by the large number of patients and the random sampling used for the dogs within these practices. The subset of the UK dog population that is unregistered with a veterinary practice was omitted from the study. Differential access to veterinary care could be breed‐related, whereby owners of some breeds may be more likely to seek veterinary intervention and therefore more likely to be included in studies such as the current. Such effects would reduce the generalisability of the current study to the wider UK dog population.

Periodontal disease is shown to be a common diagnosis in UK dogs, with one in eight dogs diagnosed annually. There are strong breed predispositions for periodontal disease, with Toy Poodle, Greyhound and Cavalier King Charles Spaniel at greatest risk while German Shepherd Dog, French Bulldog and Staffordshire Bull Terrier showed reduced risk. The study also highlights associations with conformation such as skull shape and body size. The risk of periodontal disease increases with ageing. Periodontal disease should be considered as a priority welfare concern for predisposed breeds and attention to good dental care is recommended for all dogs, especially as they age.

### Funding

This study was supported at the RVC by an award from the Kennel Club Charitable Trust and Agria Pet Insurance. Neither the Kennel Club Charitable Trust, Agria Pet Insurance nor the Kennel Club had any input in the design of the study, the collection, analysis and interpretation of data or in writing the manuscript.

### Conflict of interest

The authors have no conflicts of interest to declare.

## Supporting information


**Appendix S1.** Supporting Information.
